# ALDH2 is a novel biomarker and exerts an inhibitory effect on melanoma

**DOI:** 10.1038/s41598-024-54084-y

**Published:** 2024-02-20

**Authors:** Hua Lei, Jinfeng Liao, Xinyu Wang, Rong Huang, Chuanpeng Ying, Jianing Yang

**Affiliations:** Department of Dermatology, Sichuan Provincial People’s Hospital, University of Electronic Science and Technology of China, No. 32, West Second Section, Yihuan Road, Qingyang District, Chengdu City, 610072 Sichuan Province China

**Keywords:** Melanoma, Differently expressed genes, Hub gene, ALDH2, Biotechnology, Cancer, Cell biology, Genetics, Molecular biology

## Abstract

Melanoma is a malignant skin tumor. This study aimed to explore and assess the effect of novel biomarkers on the progression of melanoma. Differently expressed genes (DEGs) were screened from GSE3189 and GSE46517 datasets of Gene Expression Omnibus database using GEO2R. Gene Ontology and Kyoto Encyclopedia of Genes and Genomes pathway analyses were conducted based on the identified DEGs. Hub genes were identified and assessed using protein–protein interaction networks, principal component analysis, and receiver operating characteristic curves. Quantitative real-time polymerase chain reaction was employed to measure the mRNA expression levels. TIMER revealed the association between aldehyde dehydrogenase 2 (ALDH2) and tumor immune microenvironment. The viability, proliferation, migration, and invasion were detected by cell counting kit-8, 5-ethynyl-2′-deoxyuridine, wound healing, and transwell assays. Total 241 common DEGs were screened out from GSE3189 and GSE46517 datasets. We determined 6 hub genes with high prediction values for melanoma, which could distinguish tumor samples from normal samples. ALDH2, ADH1B, ALDH3A2, DPT, EPHX2, and GATM were down-regulated in A375 and SK-MEL-2 cells, compared with the human normal melanin cell line (PIG1 cells). ALDH2 was selected as the candidate gene in this research, presenting a high diagnostic and predictive value for melanoma. ALDH2 had a positive correlation with the infiltrating levels of immune cells in melanoma microenvironment. Overexpression of ALDH2 inhibited cell viability, proliferation, migration, and invasion of A375/SK-MEL-2 cells. ALDH2 is a new gene biomarker of melanoma, which exerts an inhibitory effect on melanoma.

## Introduction

Melanoma, one of lethal skin tumors cancers, stems from pigment-producing cells and also appears in other parts of the body^1–3^. Additionally, melanoma is the most aggressive and fastest-growing form of all skin cancers, which is often diagnosed at an advanced stage^4–6^. Prognosis is mainly dependent on disease stage, with 5-year survival rates for melanoma being less than 10% in patients with stage IV (distant metastatic) disease^7^. The annual incidence of melanoma has risen steadily over the past 40 years^8^. Immunotherapies, such as ipilimumab, pembrolizumab, and nivolumab, are the treatment options for malignant melanoma^9^. In addition, biomarkers associated with tumorigenesis have been identified as potential targets for molecularly targeted melanoma therapies, such as tyrosine kinase inhibitors^10^. Treatment with conventional methods suggests some defects such as poor efficacy, long treatment time, easy relapse, and drug resistance^11^. Therefore, a combination of immunotherapy and a single or multiple tyrosine kinase inhibitors has been proven to improve outcomes for melanoma patients compared to monotherapy^12^. The limitations of melanoma treatment options emphasize the need for biomarkers to guide treatment decisions^13^. Nevertheless, searching for potential gene therapeutic targets associated with melanoma is essential.

Aldehyde dehydrogenase 2 (ALDH2) is a mitochondrial enzyme, which is responsible for the metabolism of acetaldehyde and lipid peroxides^14,15^. Low expression of ALDH2 leads to the accumulation of aldehydes, which plays a key role in the progression of cancer^16^. Recent investigations prove that ALDH2 has a significant effect on cancer susceptibility^17^. In hypopharyngeal squamous cell carcinoma, ALDH2 is associated with the poorer prognosis and shorter survival of patients^18^. Additionally, down-regulation of ALDH2 contributes to the progression of hepatocellular cancer^19^. By evaluating the survival and pathological features of 455 patients with gastric cancer, it is found that overexpression of ALDH2 is related to better prognosis of patients with gastric cancer, which is reflected in the lower pathological malignancy of patients with gastric cancer with high expression of ALDH2^20^. However, the underlying molecular mechanism of ALDH2 in melanoma is still unclear and requires further investigation.

The development of analytical systems for the identification of biomarkers is an important direction in cancer therapy^21^. Numerous genes have been reported to be involved in melanoma. LRRK2 improves the survival probability of patients with cutaneous melanoma by single-cell RNA sequencing and in vitro experiments^22^. DNA binding inhibitor 1 is highly expressed in primary melanoma^23^. After examining gene expression and clinical data from cutaneous melanoma patients based on the Cancer Genome Atlas and Gene Expression Synthesis, CDCA8, DPF1, ABCC3, CAPS2, CCR6, CLU, PTK2B, SATB1, and SYNE are identified as prognostic biomarkers of melanoma, which have significant effects on the prognosis of melanoma^24^. Nevertheless, whether ALDH2 is involved in tumor regulation in melanoma has not been fully investigated.

In this study, we analyzed GSE3189 and GSE46517 datasets from Gene Expression Omnibus (GEO) database to search the differentially expressed genes (DEGs) of melanoma. After comprehensive bioinformatics analysis, ALDH2, as a key gene of melanoma was identified. Then we investigated the role of ALDH2 in proliferation, migration, and invasion of melanoma in vitro. Our research hopes to offer new perceptions into the mechanism of melanoma, which can reveal potential molecular targets for the treatment of melanoma.

## Materials and methods

### Microarray data source

The gene expression data analyzed in this study were retrieved from GEO of the National Center for Biotechnology Information (https://www.ncbi.nih.gov/geo/). Two microarray datasets, GSE3189 and GSE46517, were retrieved from the database using the search term “melanoma”.

### DEGs selection

GEO2R (www.ncbi.nlm.nih.gov/geo/geo2r) was applied to analyze the dataset. DEGs (tumor vs. control) were determined with adj. *P* < 0.05 and |logFC|≥ 2 as filter criteria. Heatmaps and volcano plots were generated to display the identified DEGs. The standardization and cross-comparison evaluation of the dataset samples were exhibited using boxplots.

### Functional enrichment analysis of common DEGs

Gene Ontology (GO) and Kyoto Encyclopedia of Genes and Genomes (KEGG) pathway enrichment analyses for common DEGs were analyzed in the DAVID (Database for Annotation, Visualization, and Integrated Discovery) database (https://david.ncifcrf.gov/). The results with the minimum *P*-value were selected to display, indicating the most significant enrichment. Bar and bubble plots were drawn to visualize the enrichment results.

### Protein–protein interaction (PPI) network construction and hub module analysis

DEGs were analyzed using the Search Tool for the Retrieval of Interacting Genes (https://www.string-db.org/) online database to reveal the interactions between proteins encoded by DEGs that played important roles in the pathogenesis of melanoma. A confidence interaction score set at 0.15 was regarded as significant standards. Subsequently, the PPI network was constructed and visualized using Cytoscape software (www.cytoscape.org/) based on the protein interaction information. and key modules were identified from the PPI network using MCODE (Molecular Complex Detection). The plugin CytoHubba (Version 0.1) of Cytoscape was employed to calculate the degree of each protein node, and the hub genes were selected according to the degree.

### Analysis of hub genes

The expression ridge plot was plotted using R package ggplot2. The expression levels of hub genes were used as variables for principal component analysis (PCA). PCA analysis was conducted to determine whether the hub genes could distinguish between the tumor samples from control samples. PC1 and PC2 were obtained as principal component variables. The value of the original variable corresponding to the arrow in the horizontal and vertical directions could reflect the correlation between the variable and PC1 and PC2, respectively.

Receiver operating characteristic (ROC) curves were plotted to evaluate the diagnostic accuracy of hub genes by calculating the area under the ROC curve (AUC) using Gene expression profile interactive analysis (http://gepia.cancer-pku.cn/). The expression of hub genes was further confirmed using the Gene Expression Profiling Interactive Analysis 2 database.

### Survival analysis and correlation analysis between key gene and immune infiltration

Kaplan–Meier plotter (http://kmplot.com/analysis/) was applied to reveal the association between the expression levels of the key gene and the survival of patients with melanoma. The correlation between the expression level of key gene and the infiltration of different types of immune cells in the melanoma microenvironment was analyzed via Tumor Immune Estimation Resource database (https://cistrome.shinyapps.io/timer/).

### Cell culturing and transfection

Human normal melanin cell line, PIG1 (American type culture collection, Manzas, Virginia, USA), and malignant melanoma cells, A375 and SK-MEL-2 cells (iCell Bioscience Inc, Shanghai, China) were maintained in Dulbecco's Modified Eagle's Medium (DMEM) supplemented with 10% fetal bovine serum at 37° C with 5% CO_2_. The pcDNA3.1-ALDH2 plasmid synthesized by GenePharma (Shanghai, China) was transfected into A375 and SK-MEL-2 cells with the presence of Lipo3000 transfection reagent (Thermo Fisher Scientific, Waltham, MA, USA). Transfected cells were harvested after 48 h of incubation in 6-well plates and the transfection efficiency was measured using quantitative real-time polymerase chain reaction (qRT-PCR).

### qRT-PCR

After TRIZOL reagent (Invitrogen, Carlsbad, CA, USA) extracting RNA from cells, the RNA concentration and absorbance values at 260 nm and 280 nm were measured using a UV spectrophotometer. Samples with an OD260/OD280 ratio between 1.9 and 2.0 indicated high purity, which was suitable for subsequent experiments. Reverse transcription was carried out using a PCR instrument to synthesize cDNA templates, and qRT-PCR was performed using ABI7500 quantitative PCR instrument (Applied Biosystems, Foster City, CA, USA). The reaction procedures were as follows: 95 °C for 30 s pre-denaturation, 95 °C for 10 s denaturation, 60 °C for 30 s annealing, and 40 cycles. GAPDH was used as an internal control. The Ct values were analyzed by the 2^−ΔΔCt^ method. The experiment was repeated three times. Primer sequences could be seen in Table 1.

**Table 1:** Primer sequences.

Name	Sequences (5′-3′)
GAPDH-F	AACTTTGGCATTGTGGAAGG
GAPDH-R	ACACATTGGGGGTAGGAACA
ALDH2-F	GACACGAGAAAGCGTCATCA
ALDH2-R	GAGAGGAGAGTGCGTGGAAC
ALDH3A2-F	TGAAGCATCCCTCCAAAATC
ALDH3A2-R	GTTGGGGCTATGTAGCGTGT
ADH1B-F	GAGTGTTGGAGAAGGGGTGA
ADH1B-R	GTGCCAAGGAAGTGGTGAAT
DPT-F	CTAGGAAGGCTGGCAGACAC
DPT-R	GGACATCACAGGAGGAGGAA
EPHX2-F	ACGACCGTGCTGAGAGAGAT
EPHX2-R	TTCAGATTAGCCCCGATGTC
GATM-F	TGAGTTTGAGCCATGCTTTG
GATM-R	TATGCATGGGATTGGGATCT

### Cell counting kit-8 (CCK-8) assay

Cell viability was detected using a Cell Counting Kit-8 (CCK-8) (Solarbio, Beijing, China). Cells were seeded in 96-well plates with 100 μL of cell suspension (1 × 10^4^/well). As directed by the manufacturer, the A375 and SK-MEL-2 cells were cultured at 37 °C until 80% confluence, and the medium was replaced with maintenance medium (2% fetal bovine serum + 98% DMEM). After adding the cell suspension, the 96-well culture plate was placed in an incubator for 24 h. After washing twice with Dulbecco’s phosphate-buffered saline, cells were added with the maintenance medium (90 μL) and 10 μL CCK-8 solution and incubated with cells for 2 h. The absorbance at 450 nm was measured using a microplate reader.

### 5-ethynyl-2′-deoxyuridine (EdU) assay

Cell proliferation was assessed using the EdU kit (Solarbio). In brief, A375 and SK-MEL-2 cells were incubated with 4% paraformaldehyde for 15 min. Afterwards, cells were permeabilized with 0.3% TritonX-100 and then stained with the reaction solution. The cells were then incubated with 4′,6-Diamidino-2-Phenylindole for 30 min in the dark. The stained cells were observed using a fluorescence microscope (Olympus, Tokyo, Japan).

### Wound healing assay

The wound-healing assay was performed to detect cell migration ability. Each experiment was repeated thrice. Briefly, the A375 and SK-MEL-2 cells (5 × 10^5^/well) were seeded in 6-well plates. After complete adherence, cells were scratched using 10 μL sterile pipette tips. The cells were washed with phosphate-buffered saline, and fresh medium was added. At 0 h, cells were photographed using an inverted microscope. Images were captured after 24 h to calculate the cell migration rate. The healing ratio (%) = (scratch spacing at 0 h − scratch spacing at 24 h)/ scratch spacing at 0 h × 100%.

### Transwell assay

In the migration assay, cell suspension (1 × 10^5^/well) was seeded into the upper chamber and 600 μl medium containing 20% fetal bovine serum was added to the lower compartment. After incubation for 24 h, cells were fixed with 4% paraformaldehyde for 30 min and stained with 0.1% crystal violet. The non-migrated cells were removed and washed three times with phosphate buffered solution. Cells were observed under a microscope to take photos for counting.

### Statistical analysis

GraphPad Prism 7.0 was used to perform a statistical analysis on the data, which were all represented as mean ± standard deviation. Comparisons between two groups were conducted by t test. One-Way ANOVA followed by Tukey's multiple comparisons test was used for comparison between multiple groups. *P* < 0.05 represented statistically significant.

### Ethics approval and consent to participate

No human and animal studies were used in this study.

## Results

### DEGs identification

**Figure 1:**
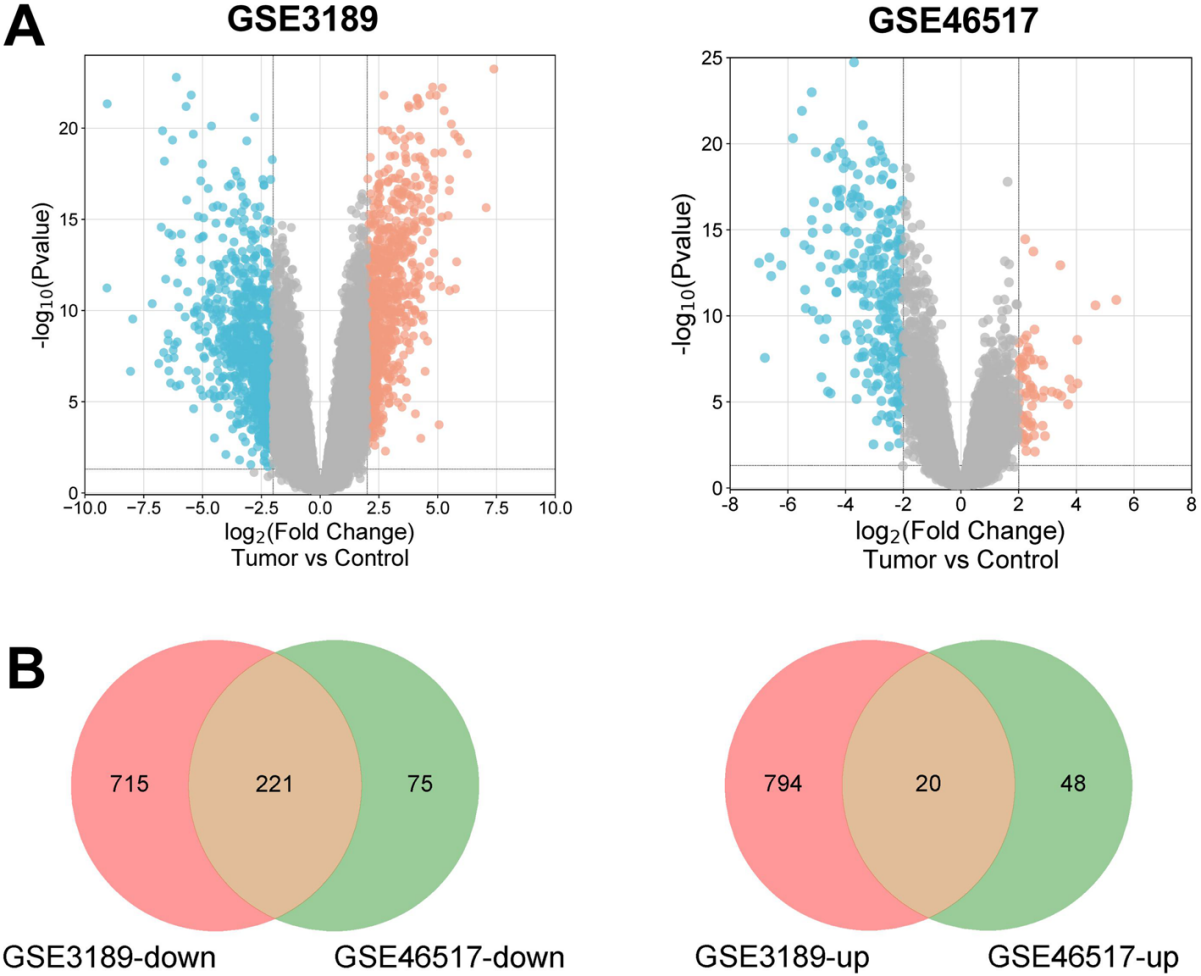
Differently expressed genes (DEGs) in GSE3189 and GSE46517 datasets. (**A**) Volcano map of DEGs in GSE3189 and GSE46517 datasets. The x-coordinate is log2FoldChange, and the y-coordinate is − log10 (*P*-value). Red dots indicate up-regulated genes and blue dots indicate down-regulated genes. (**B**) Venn diagram of common DEGs. The numbers in each circle represent the total number of DEGs, and the overlapping part of the circles represents the common DEGs.

Total 7 normal samples (Control) and 45 tumor samples of GSE3189 dataset were selected for analysis. We obtained 1750 DEGs according to adj. *P* < 0.05 and |logFC|≥ 2, of which 814 DEGs were up-regulated and 936 DEGs were down-regulated. The GSE46517 dataset included 8 normal samples (Control) and 73 tumor samples. We selected 365 DEGs in GSE46517 dataset according to adj. *P* < 0.05 and |logFC|≥ 2, of which 68 DEGs were up-regulated and 297 DEGs were down-regulated. Afterwards, the volcano maps showed the distribution of up-regulated and down-regulated DEGs in GSE3189 and GSE46517 datasets (Fig. 1A). The common DEGs in the 2 datasets were displayed in a Venn diagram, which suggested that there were 241 common DEGs between the two datasets (Fig. 1B).

Using GEO2R, the qualified samples in GSE3189 and GSE46517 datasets were obtained after data correction (Figure S1A, C). The top 25 significantly up-regulated and down-regulated DEGs (Supplementary materials) were selected to generate the heat maps of DEGs, which indicated that the sample had good clustering and high confidence (Figure S1B,D).

### Enrichment analysis of DEGs

**Figure 2:**
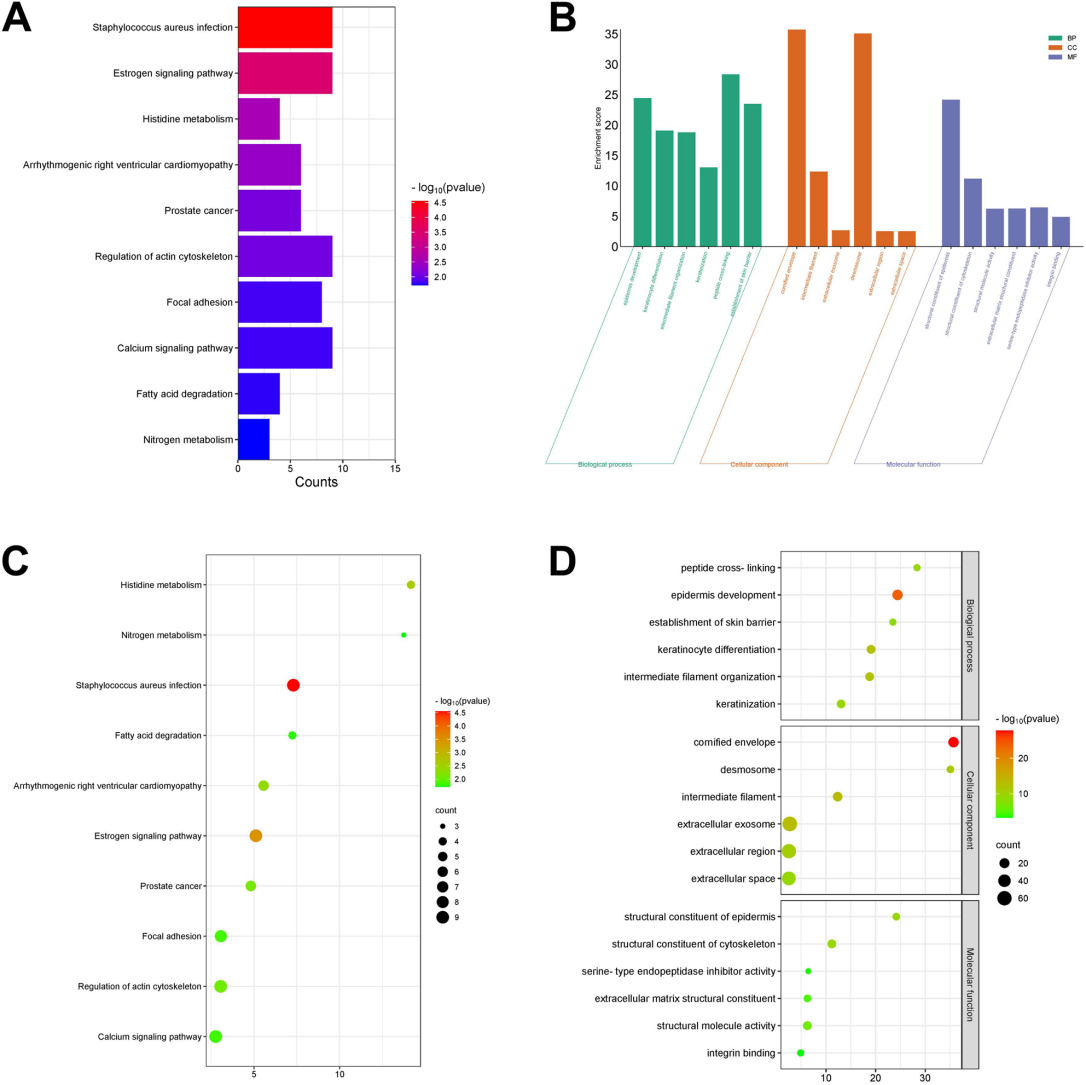
Enrichment analysis of DEGs. (**A**) Kyoto Encyclopedia of Genes and Genomes (KEGG) bar chart; (**B**) Histogram of Gene Ontology (GO) enrichment analysis. The abscissa is Go term, and the ordinate is − log10 (*P*-value). (**C**) KEGG bubble map; (**D**) GO enrichment analysis bubble map, the color depth of nodes represents the corrected p-value, and the size of nodes refers to the number of genes involved. Copyright permission: All the KEGG data in this study were publicly available from KEGG database (www.kegg.jp/kegg/kegg1.html) and we have obtained the copyright permission to use the following KEGG pathway map images in this article.

Based on the KEGG enrichment analysis, we identified the top 10 significantly enriched pathways with the minimum *P*-value, as shown in the KEGG pathway enrichment bar chart and KEGG enrichment bubble chart (Fig. 2A,C). The pathways enriched by dysregulated DEGs mainly included “Staphylococcus aureus infection”, “Estrogen signaling pathway”, “Histidine metabolism”, “Arrhythmogenic right ventricular cardiomyopathy”, “Prostate cancer”, “Regulation of actin cytoskeleton”, etc.

For the GO enrichment analysis of DEGs, the enrichment results were mainly divided into 3 categories: molecular function (MF), biological process (BP), and cellular component (CC). The top 6 significantly enriched functions were exhibited in the GO enrichment bar chart and bubble chart (Fig. 2B,D). The changes in MF enriched by DEGs were in “structural constituent of epidermis”, “structural constituent of cytoskeleton”, “structural molecule activity”, etc. The changes in BP enriched by DEGs were significantly enriched in “epidermis development”, “keratinocyte differentiation”, “intermediate filament organization”, etc. The changes in CC enriched by DEGs were mainly enriched in “cornified envelope”, “intermediate filament”, “extracellular exosome”, etc.

### Hub genes identified from PPI networks

The Search Tool for the Retrieval of Interacting Genes online database was employed to construct PPI networks based on the DEGs, which were visualized using Cytoscape software (Figure S2A). We identified the highly interconnected cluster from the PPI network of DEG as potential functional molecular complexes in melanoma (Figure S2B). Then, genes in this cluster were sequenced according to the score. After literature research, we chose 6 hub genes: ALDH2, ADH1B, ALDH3A2, DPT, EPHX2, and GATM, which were rarely reported in melanoma as the object of this research to explore their accuracy in the diagnosis and prediction of melanoma.

### Analysis of hub genes

The correlation coefficient of hub gene expression in the datasets was shown in the heat map (Figure S3A). After processing PCA analysis, the obtained PC1 and PC2 provided a variance explanation rate of 87.3%. Using PC1 and PC2 as the axes to plot the scatter plot, there was an apparent separation between the samples, further confirming the effectiveness of PC1 and PC2. There were obvious differences in the expression of hub genes between the Control group and the Tumor group, suggesting that the expression of hub genes could be a basis for distinguishing control group samples from melanoma group samples (Figure S3B). The ridge plot revealed the expression of hub genes in the GSE3189 dataset (Figure S3C).

**Figure 3:**
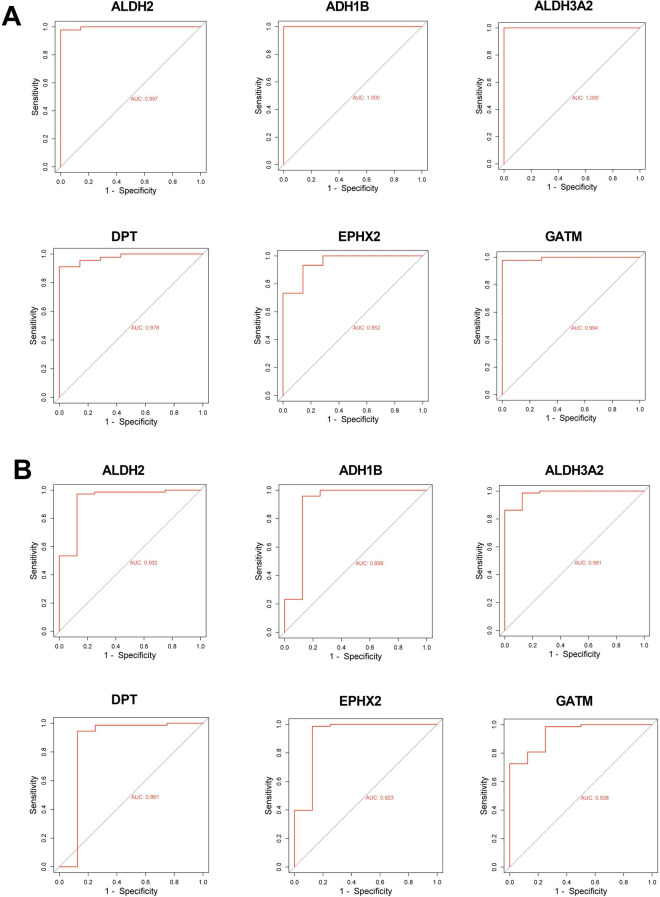
The receiver operating characteristic (ROC) analysis of hub genes. (**A**) The ROC curve plotted using the expression of hub gene in GSE3189 dataset. (**B**) The ROC curve plotted using the expression of hub gene in GSE46517 dataset. The horizontal coordinate is false positive rate and the vertical coordinate is true positive rate, indicating the expression value of the gene in the sample data.

We used the expression of ALDH2, ADH1B, ALDH3A2, DPT, EPHX2, and GATM in GSE3189 dataset to draw ROC curves, showing the false positive rates of 0.3%, 0%, 0%, 2.2%, 4.8%, 0.06%, respectively, and the true positive rate of 99.7%, 100%, 100%, 97.8%, 95.2%, 99.4%, respectively (Fig. 3A). The expression of hub genes in GSE46517 dataset was also applied to plot the ROC curve, with the false positive rates of 6.8%, 10.1%, 1.9%, 13.9%, 7.7%, 6.2%, respectively, and the true positive rate of 93.2%, 89.9%, 98.1%, 86.1%, 92.3%, 93.8%, respectively (Fig. 3B). The ROC curves indicated that the hub genes were qualified indicators for distinguishing between melanoma and healthy controls.

**Figure 4:**
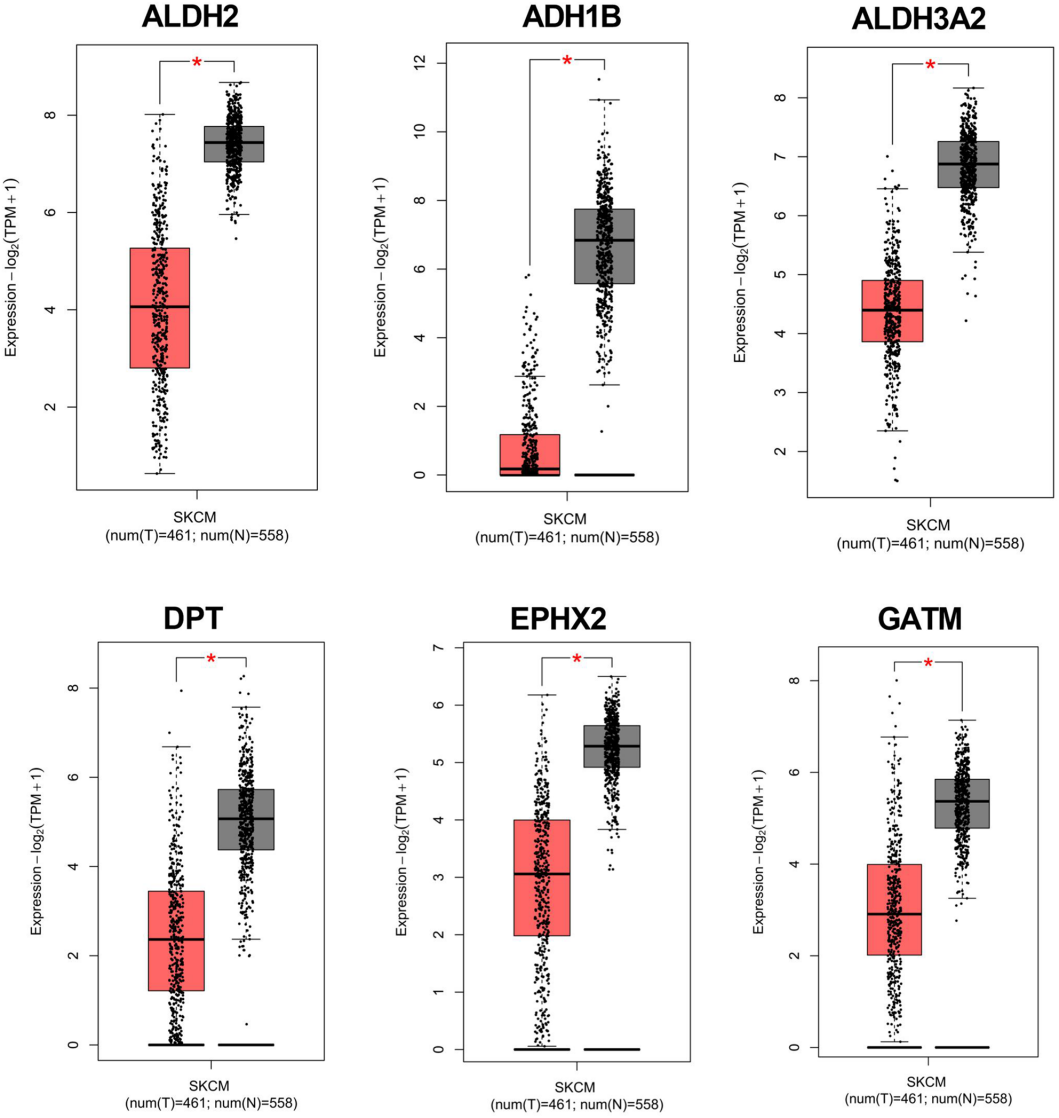
The expression levels of hub genes verified by Gene Expression Profiling Interactive Analysis 2 database. The expression levels of ALDH2, ADH1B, ALDH3A2, DPT, EPHX2, and GATM in melanoma were verified in Gene Expression Profiling Interactive Analysis 2 database.

The Expression-DIY Box-Plot function in the Gene Expression Profiling Interactive Analysis 2 database (http://gepia2.cancer-pku.cn/#index) was employed to verify the expression of the hub genes in melanoma. ALDH2, ADH1B, ALDH3A2, DPT, EPHX2, and GATM were down-regulated in the tumor tissues, which is consistent with the expression of hub genes in the GSE3189 and GSE46517 datasets (Fig. 4).

### Analysis of ALDH2 in melanoma

**Figure 5:**
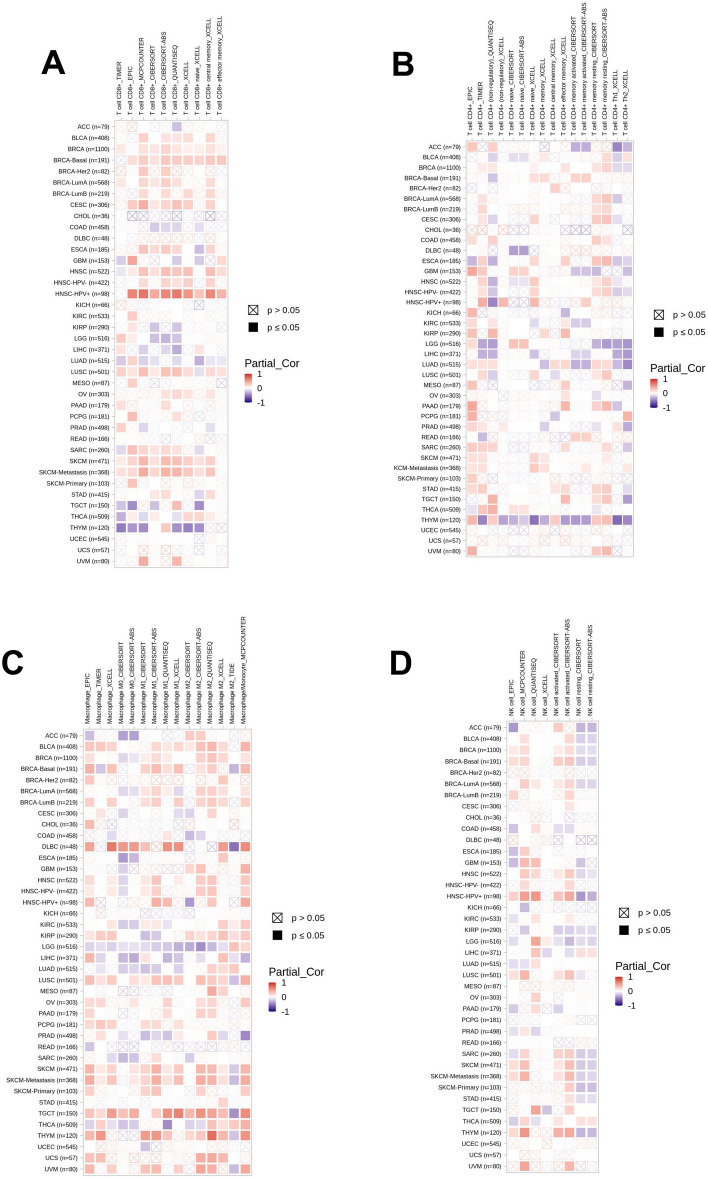
Correlation between ALDH2 and infiltrating level of immune cells. (**A**) The correlation between ALDH2 and infiltrating level of CD8+ cells. (**B**) The correlation between ALDH2 and infiltrating level of CD4+ cells. (**C**) The correlation between ALDH2 and infiltrating level of Macrophage. (**D**) The correlation between ALDH2 and infiltrating level of natural killer cells.

Survival analysis revealed a significant difference in survival rate between patients with high and low expression levels of ALDH2, which indicated the expression of ALDH2 could distinguish melanoma patients from normal people (Figure S4). The immune infiltration analysis demonstrated that the expression of ALDH2 was significantly correlated with the infiltration of the immune cells in melanoma (Figure S5). Low expression of ALDH2 had a significant impact on the infiltrating levels of immune cells, promoting immune infiltration in tumor microenvironment (Fig. 5A–D).

### Validation for the expression of hub genes using qRT-PCR

**Figure 6:**
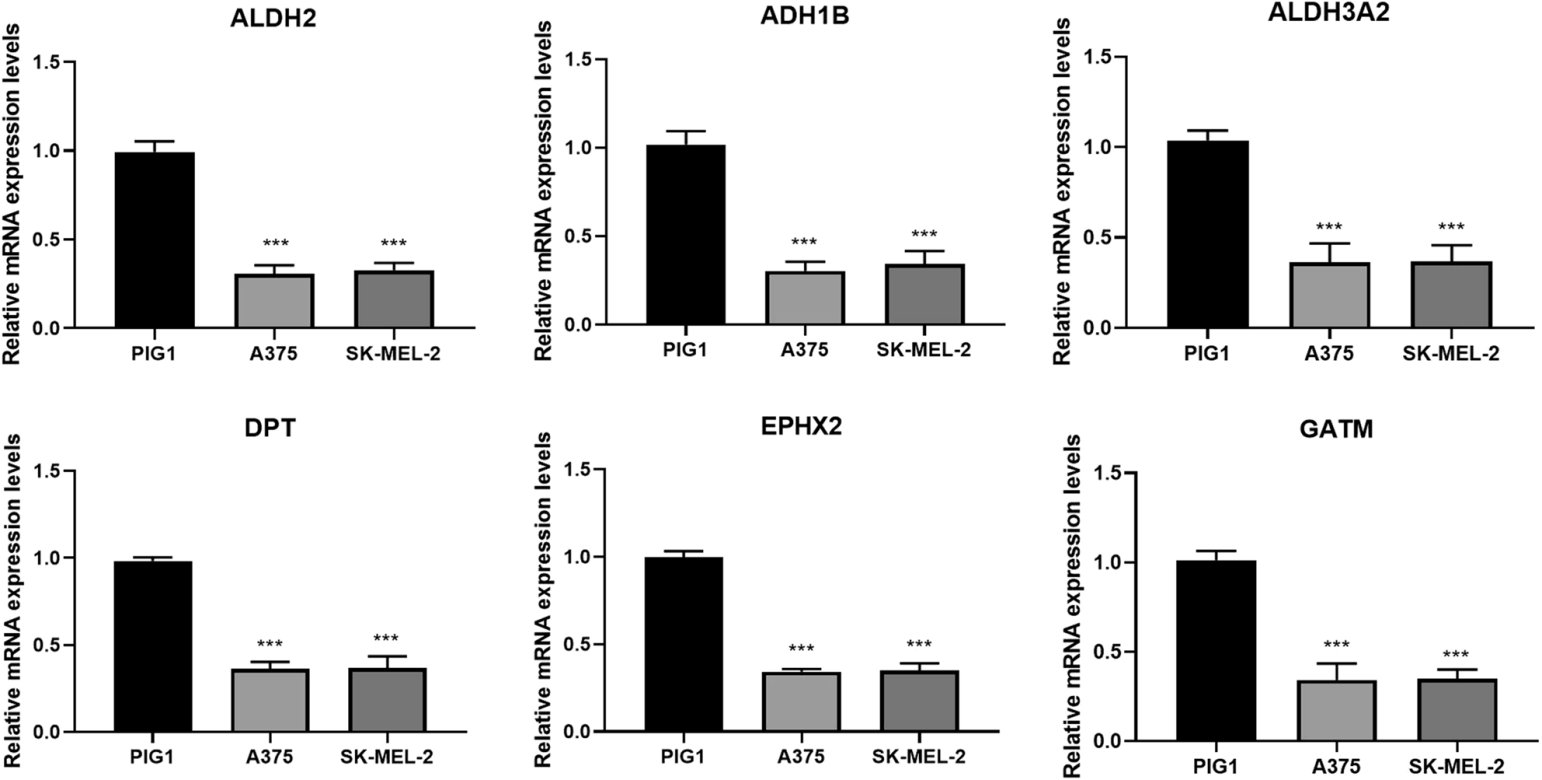
The mRNA expression of ALDH2, ADH1B, ALDH3A2, DPT, EPHX2, and GATM was measured in melanoma cells (A375/SK-MEL-2 cells) and human normal melanin cell line (PIG1 cells) by quantitative real-time polymerase chain reaction (qRT-PCR), ^**^P < 0.01, ^***^P < 0.001 versus PIG1 cells.

Compared to PIG1 cells, the mRNA expression levels of ALDH2, ADH1B, ALDH3A2, DPT, EPHX2, and GATM in A375 and SK-MEL-2 cells were significantly lower, which is consistent with the results of bioinformatics analysis (Fig. 6).

### Overexpression of ALDH2 inhibits viability, proliferation, migration, and invasion of melanoma cells

**Figure 7:**
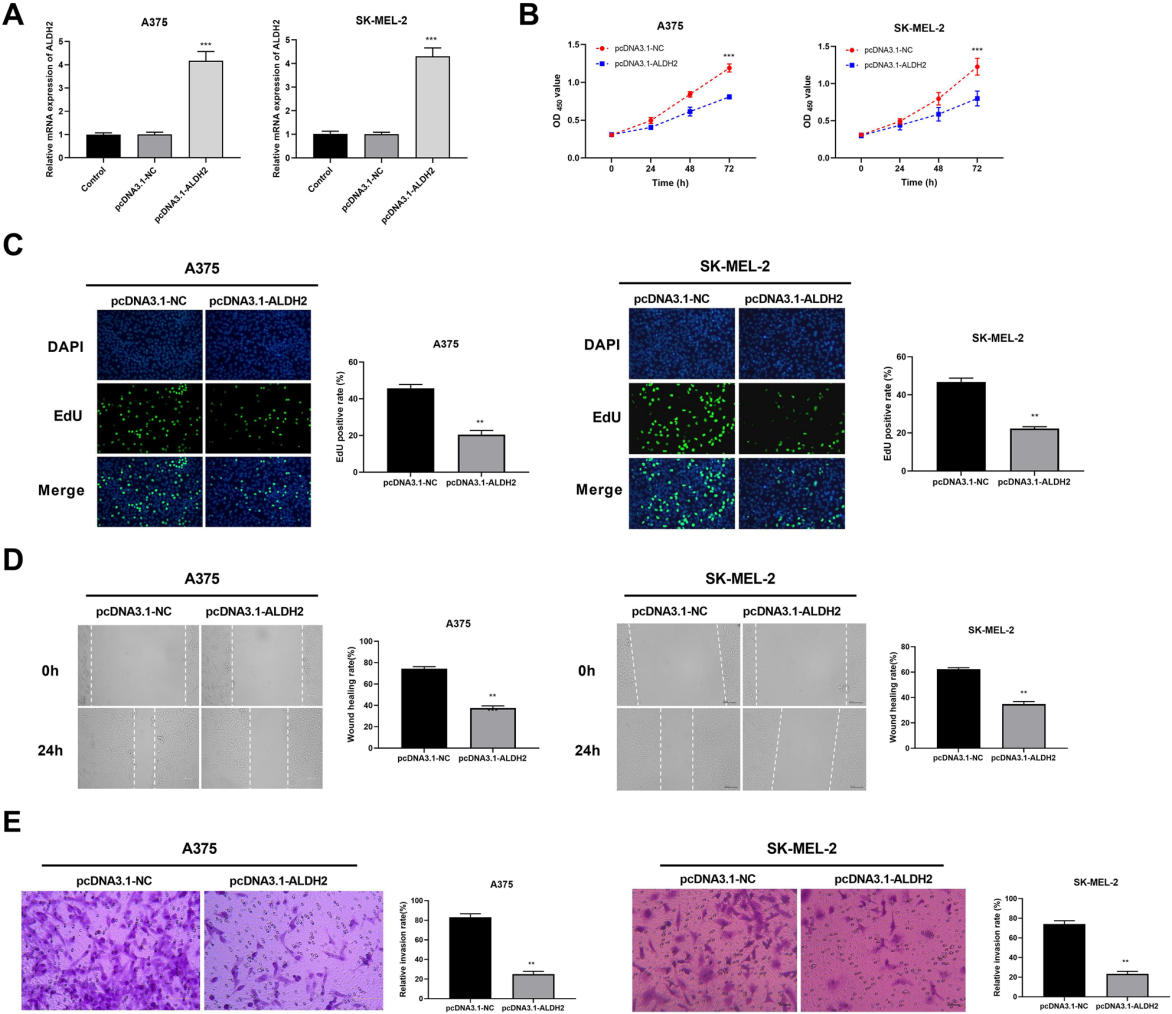
ALDH2 inhibits viability, proliferation, migration, and invasion of melanoma cells. (**A**) Transfection efficiency was detected by qRT-PCR. (**B**) The viability of A375/SK-MEL-2 cells was measured by Cell counting Kit-8 assay. (**C**) The proliferation was detected by 5-Ethynyl-2′-deoxyuridine assay. (**D**) The migration of A375/SK-MEL-2 cells was measured by wound healing assay. (**E**) The invasion of A375/SK-MEL-2 cells was measured by Transwell assay. ^***^P < 0.001 versus pcDNA3.1-NC group.

The mRNA expression of ALDH2 in the pcDNA3.1-ALDH2 group was apparently lower than that in the pcDNA3.1-NC group (Fig. 7A). The CCK-8 assay suggested that compared with the pcDNA3.1-NC group, the cell viability of A375 and SK-MEL-2 cells in the pcDNA3.1-ALDH2 group was significantly suppressed at 24 h, 48 h, and 72 h (Fig. 7B). EdU results demonstrated that the EdU-positive A375 and SK-MEL-2 cells in the pcDNA3.1-ALDH2 group were significantly reduced, compared with the pcDNA3.1-NC group (Fig. 7C). Additionally, the migration and invasion abilities A375 and SK-MEL-2 cells in the pcDNA3.1-ALDH2 group were significantly decreased, compared with the pcDNA3.1-NC group, according to wound healing and transwell assays (Fig. 7D,E).

## Discussion

Melanoma is one of the most metastatic and drug-resistant solid tumors^25^. Advances in bioinformatics tools such as sequencing and microarrays have made it possible to develop highly reliable and accurate biomarkers, which are important for early identification, rapid diagnosis, accurate prognosis, and progression prevention ^26,27^. In this paper, we identified 6 hub genes based on the gene expression dataset of GEO database and PPI network. Subsequently, we systematically assessed that hub genes were reliable and sensitive biomarkers for melanoma with high prediction values. In addition, it was determined that ALDH2 could accurately distinguish between melanoma and normal samples. Additionally, we also discovered that overexpression of ALDH2 inhibited viability, proliferation, migration, and invasion of melanoma cells.

ALDH2, ADH1B, ALDH3A2, DPT, EPHX2, and GATM have been screened out as biomarkers in various tumors based on bioinformatics analysis. Among these hub genes, ALDH2 has been reported in hepatocarcinogenesis^28^, endometrial cancer^29^, and colorectal cancer^30^. In addition, after analyzing expression profiles of GEO database, ALDH3A2 is overexpressed in gastric cancer, and survival analysis suggests that patients with low ALDH3A2 expression have significantly shorter overall survival than those with high ALDH3A2 expression, indicating that ALDH3A2 can serve as an independent predictor and a prognostic marker for gastric cancer^31^. In ovarian cancer, ADH1B is found significantly down-regulated in ovarian cancer cells and tissues after integrated bioinformatics analysis and western blot assay^32^. DPT is a candidate protein biomarker in tissue lysates of metastatic melanoma and is identified in tissue lysates of malignant melanoma by selected reaction monitoring^33^. The reduction of EPHX2 in A375 melanoma cells reveals a disruption of the antioxidant system, thus enhancing its ability to metastasize^34^. Based on the GSE45216, GSE98774, and GSE108008 datasets, GATM is identified as one of the hub genes in cutaneous squamous cell carcinoma^35^. We identified 6 hub genes of melanoma from the PPI network based on the GSE3189 and GSE46517 datasets. Taken together, ALDH2, ADH1B, ALDH3A2, DPT, EPHX2, and GATM are potential biomarkers of melanoma, which have high prediction values for melanoma.

ALDH2 is related to immune cells in various tumor microenvironments. In head and neck squamous cell carcinoma, ALDH2 is associated with CD8+ T cell infiltration in the tumor microenvironment^36^. As a biomarker of prostate cancer prognosis, ALDH2 expression is positively associated with B cells, CD8+ T cells, neutrophils, and macrophages^37^. ALDH2 expression level is significantly lower in hepatocellular carcinoma tissue than in normal liver tissue and is correlated with the immune infiltration of dendritic cells and macrophages^38^. In lung adenocarcinoma, ALDH2 is highly associated with B cell activity and partial correlation with CD4+ T-cell activity, suggesting that ALDH2 interacts with B cells and T cells in patients with lung adenocarcinoma^39^. Herein, we found that the expression of ALDH2 was correlated with CD8+ , CD4+, macrophage and other immune cells, which could promote the infiltration of immune cells. All in all, ALDH2 was associated with tumor immune infiltration of immune cells in tumor microenvironment of melanoma.

The role of ALDH2 in tumorigenesis, growth, and metastasis has been widely reported. Low or high expression of ALDH2 promotes or inhibits tumor progression in various cancers^40^. ALDH2 expression is down-regulated in hepatocellular carcinoma, which can be used as a tumor inhibitor, and overexpression of ALDH2 inhibits the proliferation, migration, and invasion in hepatocellular cancer^41^. Knockdown of ALDH2 expression can significantly suppress the migration of colon cancer cells, while activation of ALDH2 can promote the migration of colon cancer cells^42^. In lung adenocarcinoma, down-regulation of ALDH2 expression leads to the accumulation of acetaldehyde, which promotes the proliferation and migration abilities of lung adenocarcinoma cells^43^. The expression of ALDH2 in human lung cancer is significantly down-regulated, and the overexpression of ALDH2 causes an inhibitory effect on the proliferation and colony formation of lung cancer cells, while the silencing of ALDH2 can reverse the tumor-suppressive effects^44^. Our study found that overexpression of ALDH2 suppressed the viability, proliferation, migration, and invasion of A375 and SK-MEL-2 cells. Taken together, overexpression of ALDH2 may exert an anti-tumor function in melanoma.

In brief, we identified a key gene biomarker, ALDH2, after comprehensive bioinformatics analysis, which had good specificity and sensitivity to be a biomarker of melanoma. ALDH2 was down-regulated in melanoma, promoting the infiltration levels of immune cells in tumor microenvironment. Most importantly, overexpression of ALDH2 suppressed the viability, proliferation, migration, and invasion of melanoma cells, which may contribute to the anti-tumor outcomes in the development of melanoma. We provided a new insight into the molecular mechanisms and a potential biomarker for diagnosis of melanoma. Our findings indicate that overexpressing ALDH2 may be a potential gene target for the therapies for melanoma.

### Supplementary Information

Supplementary Figures.

Supplementary Information 1.

Supplementary Information 2.

Supplementary Information 3.

## Data Availability

The datasets used and/or analysed during the current study are available from the corresponding author on reasonable request.
