# Supplementary material for: ALDH2 is a novel biomarker and exerts an inhibitory effect on melanoma
Source: Sci Rep. 2024 Feb 20;14:4183. doi: 10.1038/s41598-024-54084-y (PMC10879513; doi:10.1038/s41598-024-54084-y)

**Figure S1** A: Correction results of samples in GSE3189 dataset. B: Heatmap of DEGs in GSE3189 dataset. C: Correction results of samples in GSE46517 dataset. D: Heatmap of DEGs in GSE46517 dataset. Heat map: Horizontal representation of genes, each column is a sample, red represents high expression genes, blue represents low expression genes.


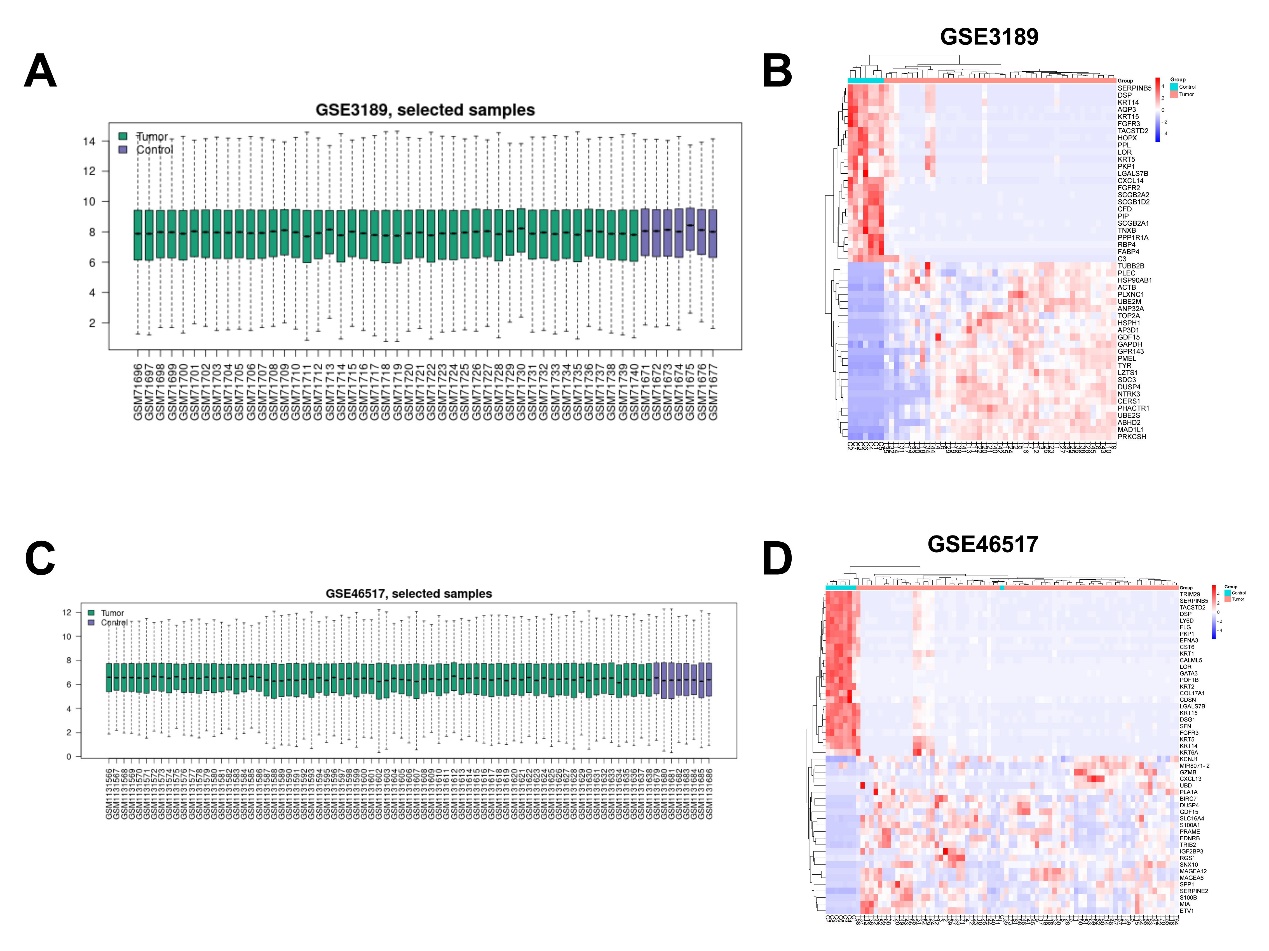


**Figure S2:** A: Protein-protein interaction network; B: Hub genes identified based on MCODE analysis. The lines between the nodes represent the interactions between genes.
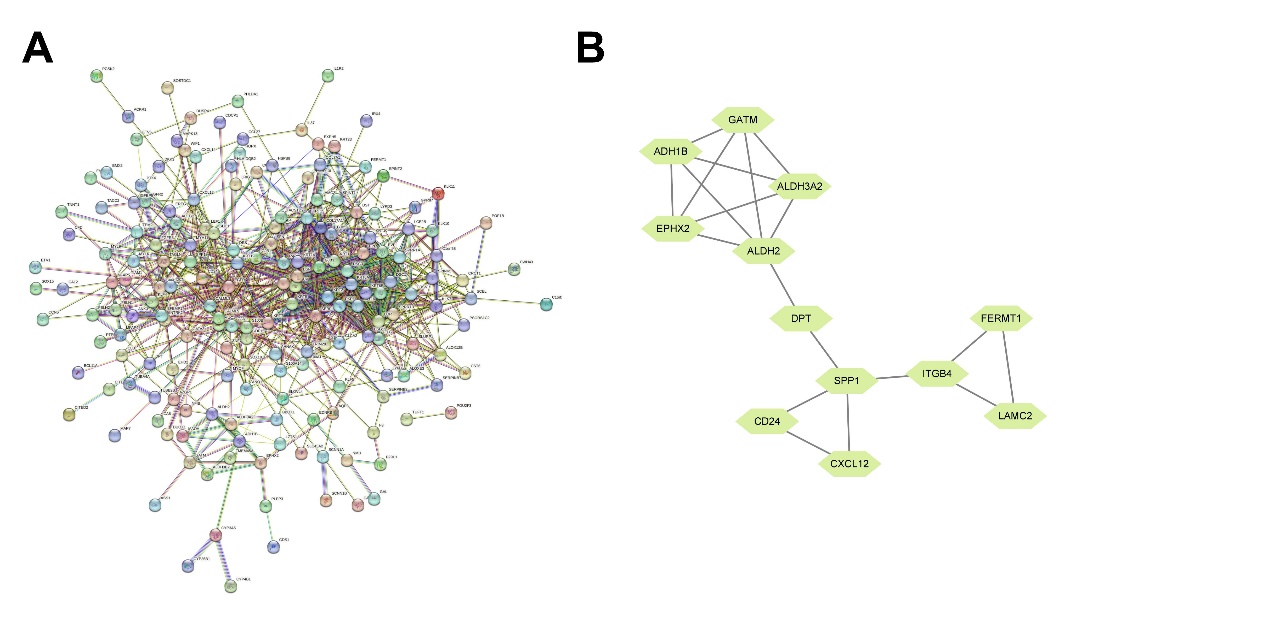


**Figure S3** Analysis of hub genes A: The correlation coefficient of hub gene expression in the datasets. B: Principal component analysis of hub gene. Axes of PC1 and PC2 in the figure are the first and second principal components (i.e., the explanation rate of differences by potential variables); Dots represent samples, and different colors represent different groups. C: The expression of ridge map of hub genes. The horizontal coordinate is gene expression, the shape of the mountain represents the dispersion between a set of data, and the height is the number of samples corresponding to the gene expression.


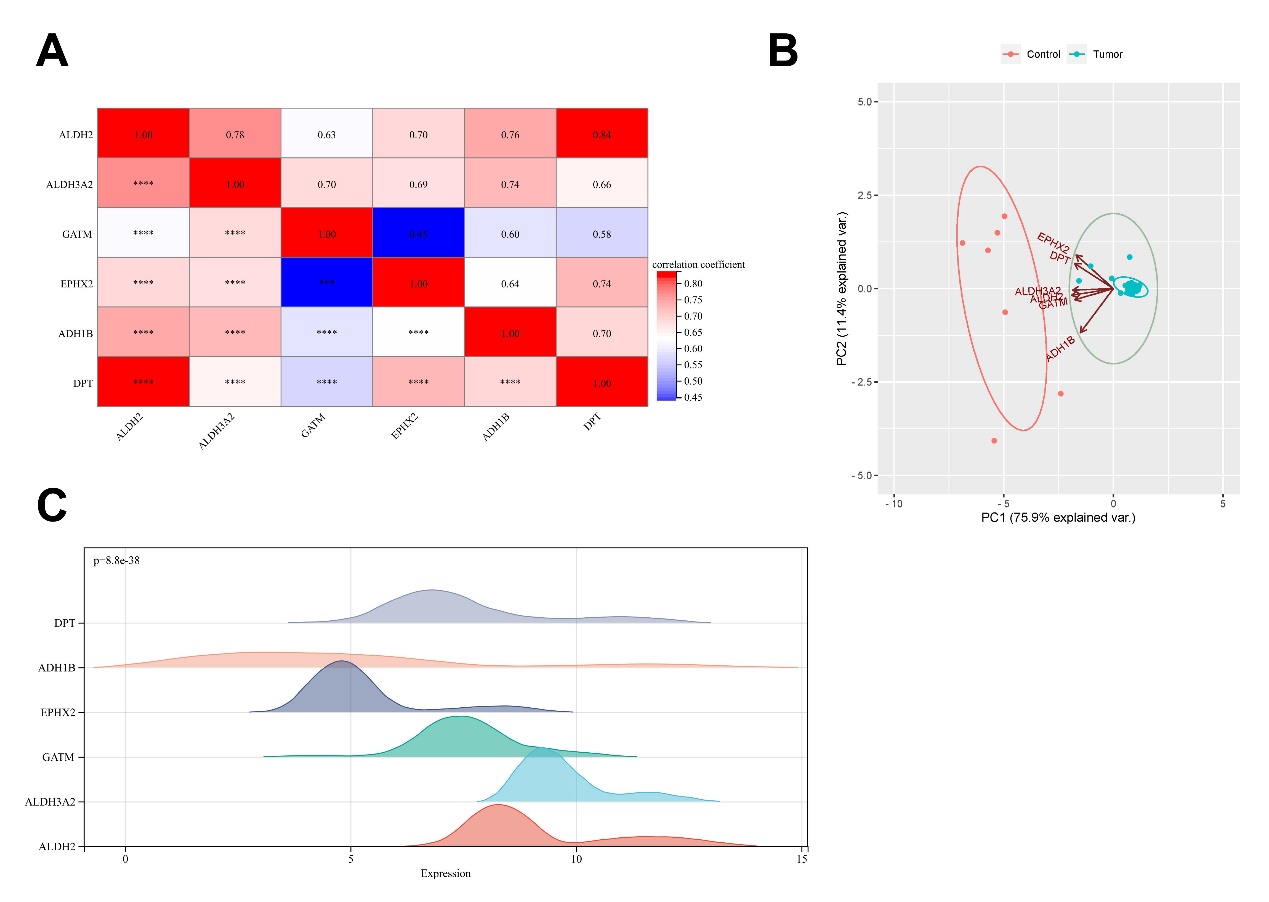


**Figure S4** The Gene Expression Profiling Interactive Analysis 2 database verified that the difference of ALDH2 expression had significant differences in the prognosis of SKCM patients.


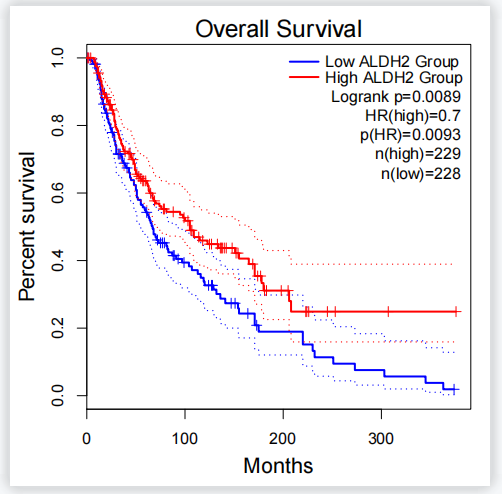


**Figure S5** Immune infiltration of ALDH. The coloration of ALDH2 in melanoma cells and infiltration of B cell, CD8+T cell, CD4+T cell, Macrophage, Neutrophil, Dendritic cell.
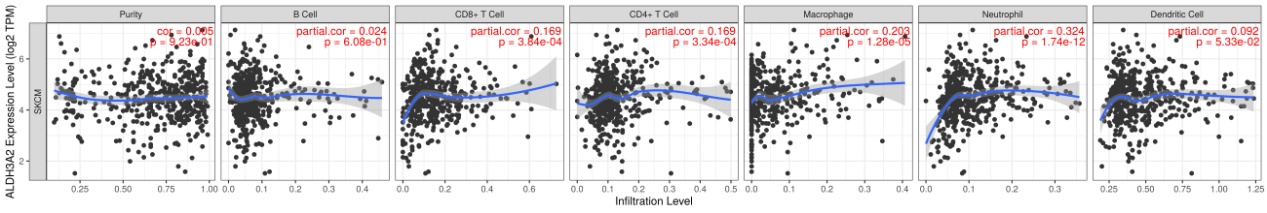

Supplement: Supplementary file 1 — Supplementary Figures. [file 41598_2024_54084_MOESM1_ESM.docx]
